# Impacto do Cardioversor Desfibrilador Implantável na Vida de seus Portadores

**DOI:** 10.36660/abc.20240196

**Published:** 2024-06-14

**Authors:** Silas dos Santos Galvão

**Affiliations:** 1 Centro Avançado de Ritmologia e Eletrofisiologia Beneficência Portuguesa de São Paulo São Paulo SP Brasil Centro Avançado de Ritmologia e Eletrofisiologia (C.A.R.E.) - Beneficência Portuguesa de São Paulo, São Paulo, SP – Brasil

**Keywords:** Arritmias Cardíacas, Desfibriladores Implantáveis/tendências, Morte Súbita Cardíaca, Qualidade de Vida, Perfil de Impacto da Doença

O primeiro implante de CDI em humanos ocorrido em 1980,^1^ seguido da incorporação desse dispositivo ao arsenal terapêutico da cardiologia no início dos anos 90, foi um dos maiores avanços no tratamento das arritmias cardíacas. Sendo a terapêutica de eleição com evidências de eficácia para grande número de pacientes com risco de morte súbita cardíaca de causas não reversíveis,^2^ os CDIs apresentam algumas peculiaridades que podem impactar na vida cotidiana de seus portadores. No início, os CDIs eram dispositivos enormes, somente passíveis de serem implantados a nível abdominal, com seus eletrodos e palhetas para aplicação do choque implantados diretamente no coração mediante toracotomia. Nesse momento, o impacto desses dispositivos na vida de seus portadores era grande, desde estético, até pelo grande número de choques inapropriados, causados por algoritmos ainda precários de discriminação da arritmia a ser tratada: taquicardia ventricular (TV) ou fibrilação ventricular (FV), além das primeiras programações ainda inadequadas. Em nossa experiência inicial com CDIs no final dos anos 80 e início dos anos 90, tivemos muitos problemas de aceitação, sendo que alguns pacientes solicitaram até mesmo retirar o dispositivo após quadro de choques de repetição. Com a espetacular evolução que sofreram, os CDIs incorporaram todos os avanços tecnológicos, melhorando sua performance diagnóstica na liberação das terapêuticas e passando a ter dimensões pouco maiores que os marcapassos, propiciando implantes dos dispositivos a nível torácico, com eletrodos totalmente transvenosos por cirurgia minimamente invasiva, dispensando a toracotomia. Não obstante esses avanços, uma parte considerável dos pacientes persiste referindo impacto na sua qualidade de vida. Isso se deve principalmente a um aspecto: o CDI é o único dispositivo cardíaco eletrônico implantável cujo funcionamento normal terapêutico é percebido pelo paciente, com sensações descrita pela grande maioria como muito desagradáveis, especialmente a terapia com choque,^3^ sendo a pior situação quando ocorrem choques de repetição. O aperfeiçoamento dos algoritmos diagnósticos das taquiarritmias, e o maior conhecimento da programação dos dispositivos, reduziram consideravelmente os choques inapropriados,^4^ entretanto os choques apropriados, mesmo os de repetição conhecidos como tempestades elétricas persistem ocorrendo,^5,6^ já que correspondem ao funcionamento normal do aparelho na reversão de parada cardíaca por FV, ou de arritmia potencialmente maligna como a TV. Felizmente, hoje esses graves episódios são bem menos frequentes e passíveis de serem tratados por meio de medicamentos e/ou procedimentos intervencionistas como ablação por cateter.^7^

O artigo "Preditores de Qualidade de Vida, Ansiedade e Aceitação em Pacientes com Cardioversor-Desfibrilador Implantável"^8^ avaliou esses aspectos em um número razoável de pacientes submetidos ao primeiro implante ou troca de gerador de CDI, mostrando que cerca de um quarto dos pacientes apresentaram algum tipo de impacto negativo. Mostrou também que a idade de 60 anos ou mais, a ausência de fibrilação atrial (FA) e o sexo feminino, foram preditores de melhores resultados. Essas informações são importantes e merecem algumas considerações: 1 - Tendo melhorado consideravelmente ao longo dos anos, o CDI ainda impacta negativamente na vida de um número relativamente importante de seus portadores. Isso estimula a melhora tecnológica dos dispositivos otimizando as terapias, além da melhor abordagem e preparo psicológico do paciente no pré-operatório. 2 - Os piores resultados dos pacientes mais jovens podem ser explicados pela sensação de grande limitação das atividades impostas aos portadores de CDI, entretanto essas limitações são devidas mais a cardiopatia de base do que propriamente ao dispositivo. Em alguns casos, sem maiores alterações da função cardiológica, até mesmo atividades esportivas profissionais de impacto podem ser liberadas,^9,10^ como no caso de 2 jogadores de futebol que disputaram a última copa do mundo portando CDI. 3 - No caso dos portadores de FA, essa arritmia muitas vezes sintomática, não infrequentemente está relacionada a cardiopatias com pior função cardiológica, o que aumenta as limitações. Além disso a FA pode ser responsável por choques inapropriados, passíveis de serem evitados com programação adequada. 4 - Em relação ao fato de as mulheres apresentarem melhores resultados apesar dos problemas estéticos que esses dispositivos podem causar, isso se deve provavelmente a maior tolerância às terapias e a melhor compreensão das possíveis limitações de suas atividades, por serem portadores de CDI.

A qualidade de vida dos portadores de CDI depende de vários aspectos, sendo os mais importantes: o conhecimento por parte do paciente de sua cardiopatia e dos riscos a que está sujeito, do tipo e forma da aplicação das terapias e seus resultados. Nesse sentido o médico especialista responsável é fundamental no esclarecimento desses aspectos e de todas as dúvidas que possa haver, além de procurar transmitir enfaticamente a sensação de segurança e proteção que esses dispositivos podem trazer. Em algumas situações de maior complexidade, pode ser necessário abordagem multidisciplinar, com a participação de psicólogos e/ou psiquiatras. Nesse sentido o artigo citado contribui para a identificação dos grupos mais vulneráveis a essas situações.

Apesar desses problemas, o CDI é um dispositivo eletrônico implantável dos mais importantes, sendo indispensável no arsenal terapêutico da cardiologia, com sua capacidade de salvar vidas largamente comprovada por inúmeros estudos científicos abrangentes.
