# Supplementary material for: Impacto do Cardioversor Desfibrilador Implantável na Vida de seus Portadores
Source: Arq Bras Cardiol. 2024 Jun 14;121(4):e20240196. [Article in Portuguese] doi: 10.36660/abc.20240196 (PMC11216330; doi:10.36660/abc.20240196)
Supplement: Translation

# Translation: Impact of the Implantable Cardioverter Defibrillator on the Lives of its Patients

**DOI:** 10.36660/abc.20240196i

**Authors:** Silas dos Santos Galvão

**Affiliations:** 1 São Paulo SP Brazil Centro Avançado de Ritmologia e Eletrofisiologia (C.A.R.E.) - Beneficência Portuguesa de São Paulo, São Paulo, SP – Brazil

**Keywords:** Arrhythmias, Cardiac, Defibrillators, Implantable/trends, Death, Sudden, Cardiac, Quality of Life, Sickness Impact Profile

The first ICD implant in humans occurred in 1980,^1^ followed by the incorporation of this device into the therapeutic arsenal of cardiology in the early 1990s, it was one of the greatest advances in the treatment of cardiac arrhythmias. Being the therapy of choice with evidence of efficacy for a large number of patients at risk of sudden cardiac death from non-reversible causes,^2^ ICDs have some peculiarities that can impact the daily lives of their carriers. Initially, ICDs were huge devices, only capable of being implanted at the abdominal level, with their electrodes and reeds for delivering the shock implanted directly into the heart through thoracotomy. At that time, the impact of these devices on the lives of their users was great, from aesthetics to the large number of inappropriate shocks, caused by still precarious algorithms for discriminating the arrhythmia to be treated: ventricular tachycardia (VT) or ventricular fibrillation (VF), in addition to the first programs that were still inadequate. In our initial experience with ICDs in the late 1980s and early 1990s, we had many acceptance problems, with some patients even requesting to remove the device after experiencing repeated shocks. With the spectacular evolution they have undergone, ICDs have incorporated all technological advances, improving their diagnostic performance in the release of therapies and now having dimensions slightly larger than pacemakers, allowing device implants at the thoracic level, with fully transvenous electrodes through minimally invasive surgery, eliminating thoracotomy. Despite these advances, a considerable number of patients continue to report an impact on their quality of life. This is mainly due to one aspect: the ICD is the only implantable electronic cardiac device whose normal therapeutic functioning is perceived by the patient, with sensations described by the vast majority as very unpleasant, especially shock therapy,^3^ the worst situation being when repeated shocks occur. The improvement of diagnostic algorithms for tachyarrhythmias, and greater knowledge of device programming, have considerably reduced inappropriate shocks,^4^ however, appropriate shocks, even repetitive shocks known as electrical storms, continue to occur,^5,6^ as they correspond to the normal functioning of the device in reversing cardiac arrest due to VF, or potentially malignant arrhythmia such as VT. Fortunately, today these serious episodes are much less frequent and can be treated through medication and/or interventional procedures such as catheter ablation.^7^

The article "Predictors of Quality of Life, Anxiety and Acceptance in Patients with Implantable Cardioverter-Defibrillator"^8^ evaluated these aspects in a reasonable number of patients undergoing the first implantation or replacement of an ICD generator, showing that around a quarter of the patients presented some kind of negative impact. It also showed that being 60 years old or older, the absence of atrial fibrillation (AF), and being female were predictors of better results. This information is important and deserves some consideration: 1- Having improved considerably over the years, ICD still negatively impacts the lives of a relatively important number of its sufferers. This encourages the technological improvement of devices, optimizing therapies, in addition to better approach and psychological preparation of the patient pre-operatively. 2- The worse results of younger patients can be explained by the feeling of great limitation of activities imposed on those with ICDs, however, these limitations are due more to the underlying heart disease than to the device itself. In some cases, without major changes in cardiological function, even impactful professional sporting activities can be allowed,^9,10^ as in the case of two football players who played in the last World Cup with ICD. 3- In the case of people with AF, this arrhythmia, which is often symptomatic, is not infrequently related to heart disease with worse cardiological function, which increases limitations. Furthermore, AF may be responsible for inappropriate shocks, which can be avoided with adequate programming. 4- Regarding the fact that women present better results despite the aesthetic problems that these devices can cause, this is probably due to greater tolerance to therapies and a better understanding of the possible limitations of their activities, as they have an ICD.

The quality of life of ICD patients depends on several aspects, the most important of which are: the patient's knowledge of their heart disease and the risks to which they are subject, the type and form of application of therapies, and their results. In this sense, the responsible specialist doctor is essential in clarifying these aspects and any doubts that may exist, in addition to emphatically seeking to convey the feeling of safety and protection that these devices can bring. In some more complex situations, a multidisciplinary approach may be necessary, with the participation of psychologists and/or psychiatrists. In this sense, the article cited contributes to the identification of the groups most vulnerable to these situations.

Despite these problems, the ICD is one of the most important implantable electronic devices, being indispensable in the therapeutic arsenal of cardiology, with its ability to save lives largely proven by numerous comprehensive scientific studies.
